# First-line antihypertensive treatment in patients with pre-diabetes: Rationale, design and baseline results of the ADaPT investigation

**DOI:** 10.1186/1475-2840-7-22

**Published:** 2008-07-24

**Authors:** Walter Zidek, Joachim Schrader, Stephan Lüders, Stephan Matthaei, Christoph Hasslacher, Joachim Hoyer, Peter Bramlage, Claus-Dieter Sturm, W Dieter Paar

**Affiliations:** 1 Medical Department IV, University Hospital Charité, Campus Benjamin-Franklin, Berlin, Germany; 2 St. Joseph's Hospital and INFO GmbH Institute for Hypertension and Cardiovascular Research, Cloppenburg, Germany; 3 Diabetes Center, Quakenbrück, Germany; 4 Department for Internal Medicine, St. Joseph's Hospital, Heidelberg, Germany; 5 Clinic for Internal Medicine, Nephrology, Marburg, Germany; 6 Institute for Clinical Pharmacology, Medical Faculty Carl Gustav Carus, TU Dresden, Germany; 7 Medical Department, Sanofi-Aventis Germany, Berlin, Germany

## Abstract

**Background:**

Recent clinical trials reported conflicting results on the reduction of new-onset diabetes using RAS blocking agents. Therefore the role of these agents in preventing diabetes is still not well defined. Ramipril is an ACE inhibitor (ACEi), that has been shown to reduce cardiovascular events in high risk patients and post-hoc analyses of the HOPE trial have provided evidence for its beneficial action in the prevention of diabetes.

**Methods:**

The ADaPT investigation ("ACE inhibitor-based versus diuretic-based antihypertensive primary treatment in patients with pre-diabetes") is a 4-year open, prospective, parallel group phase IV study. It compares an antihypertensive treatment regimen based on ramipril versus a treatment based on diuretics or betablockers. The primary evaluation criterion is the first manifestation of type 2 diabetes. The study is conducted in primary care to allow the broadest possible application of its results. The present article provides an outline of the rationale, the design and baseline characteristics of AdaPT and compares these to previous studies including ASCOT-BLPA, VALUE and DREAM.

**Results:**

Until March 2006 a total of 2,015 patients in 150 general practices (general physicians and internists) throughout Germany were enrolled. The average age of patients enrolled was 67.1 ± 10.3 years, with 47% being male and a BMI of 29.9 ± 5.0 kg/m^2^. Dyslipidemia was present in 56.5%. 37.8% reported a family history of diabetes, 57.8% were previously diagnosed with hypertension (usually long standing). The HbA1c value at baseline was 5.6 %. Compared to the DREAM study patients were older, had more frequently hypertension and patients with cardiovascular disease were not excluded.

**Conclusion:**

Comparing the ADaPT design and baseline data to previous randomized controlled trial it can be acknowledged that AdaPT included patients with a high risk for diabetes development. Results are expected to be available in 2010. Data will be highly valuable for clinical practice due to the observational study design.

## Background

Hypertension is the leading cause of morbidity and mortality worldwide [1]. The concomitant manifestation of type 2 diabetes mellitus leads to a substantial further increase in risk [2,3]. While about 50% of patients in German primary care were hypertensive in a recent cross-sectional survey, 12% of all patients had a co-manifestation of hypertension and diabetes [4].

Not only hypertensive patients with diabetes, but also hypertensive patients without diabetes tend to be resistant to insulin stimulated glucose uptake and are hyperinsulinaemic compared with normotensive controls [5]. About 20% of patients with hypertension will develop type 2 diabetes in a three year period [6] and new onset diabetes in treated hypertensive patients is not trivial as recent studies suggest [7,8].

The risk for subsequent cardiovascular (CV) disease in patients with pre-diabetes is not different from those who had both hypertension and diabetes already at baseline [9]. The adjusted relative risk of events was about 3-times higher in both previous and new onset diabetes compared to patients with hypertension but without diabetes [9].

### Antihypertensive drugs and new-onset diabetes

The roles of antihypertensive agents and in particular those that inhibit the RAS in the acceleration or deceleration of diabetes manifestation have been discussed controversial and study results on this question are not consistent.

The RAS itself plays a pivotal role in the development of diabetes. Over-activity appears to be linked to reduced insulin and glucose delivery to the peripheral skeletal muscle and impaired glucose transport and response to insulin signalling pathways, thus increasing insulin resistance [10]. Activation of a local pancreatic RAS, in particular within the islets, may represent an independent mechanism for the progression of islet cell damage in diabetes. In fact, impaired pancreatic islet function may predominate quantitatively over peripheral insulin resistance in impaired glucose tolerance [11].

Drugs that interrupt the RAS like angiotensin converting enzyme inhibitors (ACEi) and angiotensin receptors blockers (ARBs) are likely to be beneficial in the prevention of diabetes [10,12]. A series of recent large-scale prospective randomised studies of 3–6 year duration such as CAPP, INSIGHT, LIFE or ALLHAT, reported a remarkably consistent reduction in the incidence of type 2 diabetes in hypertensive patients reported with either ACEi-based or ARB-based therapy (reviewed by Jandeleit-Dahm in [10]). The comparator groups were based on thiazide-diuretics, β-blockers, the calcium channel blocker amlodipine or placebo, respectively.

**Figure 1:**
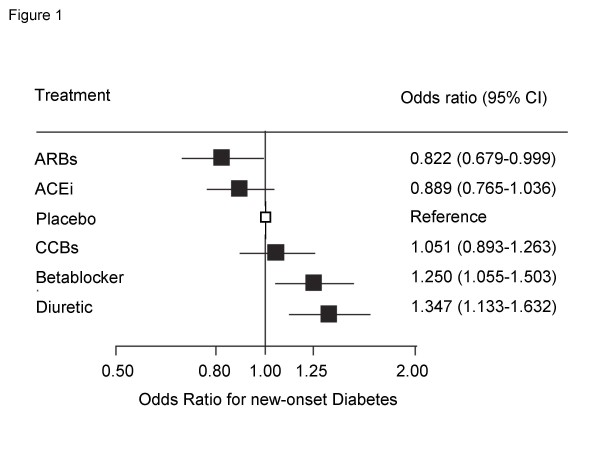
Diabetes incidence – results of full Bayesian network meta-analysis of 22 trials with 143153 patients [14], modified from [15].

In a large meta-analyis, Abuissa et al. calculated the average risk reduction in 6 of these trials using ACEi and 7 trials using ARBs. The reduction of new onset-diabetes was 24% for ACEi, 23% for ARBs and 23% for the combined data-set [13]. Furthermore a recent network meta-analysis of randomized controlled trials showed that while patients taking betablockers and diuretics show an increased incidence of diabetes, it is reduced in patients using ACEi or ARBs (Figure 1) [14,15].

Endpoint studies to elucidate the role of antihypertensive agents on new onset diabetes related morbidity and mortality are however scarce. The VALUE trial with valsartan was the only trial to include new-onset diabetes as a pre-specified endpoint [16]. Patients were normoglycemic, those with abnormal glucose values were excluded. While 16.4% of patients in the amlodipine arm (up to 10 mg) developed diabetes over a mean follow-up of 4.2 years, 13.1% developed such in the valsartan arm (up to 160 mg); p < 0.0001. The ASCOT-BPLA study, which was a randomised controlled trial of the prevention of CHD and other vascular events by BP and cholesterol lowering in a factorial study design, was prematurely stopped in December 2004 [17,18]. The study was designed to resolve whether newer antihypertensive strategies that use calcium channel blockers (CCBs) and ACEi are superior to older treatments using betablockers and diuretics. As a key finding there was a substantial excess of new diabetes (increase of 30%) in the beta blocker/diuretic arm [17]. DREAM investigated the effect of ramipril (up to 15 mg) compared to placebo [6]. In this randomised controlled trial ramipril significantly increased regression to normoglycemia in patients with impaired glucose tolerance. It did however not influence the risk of a combined endpoint consisting of new-onset diabetes or death over a 3 year observational period. Interpretation of DREAM is limited by a number of details: 1) hypertension was not an inclusion criterion (mean blood pressure at baseline 136/83 mmHg), 2) comparison was made to placebo instead of diuretics or betablockers (which would be reasonable based on the analysis of Elliott [15]) and 3) betablockers were allowed in both the ramipril and placebo groups.

### Rationale for ADaPT

Thus, despite the strong evidence for a reduction of new-onset diabetes from several RCTs and meta-analyses, there is an ongoing controversy about the clinical significance, the comparability of agents within one drug class, or the generalisability of these findings into clinical practice [19-21]. The "*ACE inhibitor-based versus diuretic-based antihypertensive primary treatment in patients with prediabetes*" (ADaPT) study addresses this issue. On the basis of the existing body of evidence, it appeared likely that patients with impaired fasting glucose (IGF) according to the screening on pre-diabetes (PreDiSc Score) will benefit from tight blood pressure control and further effects from RAS-inhibition by the ACEi ramipril in terms of manifestation of type 2 diabetes. The long-term outcomes of this treatment regimen will be compared to a regimen based on diuretics and/or β-blockers.

## Materials and methods

### Design

The ADaPT investigation is a comparative Post Marketing Surveillance according to §67(6) German Drug Law, performed by the German Hypertension League. It was designed as an open, prospective, non-randomised parallel group observational investigation in 150 general practices (general physicians and internists) throughout Germany.

### Patient population

#### Inclusion criteria

Patients eligible for this study were at high risk for the development of type 2 diabetes according to the modified *PreDiSc *Score [22]: They had to be ≥ 45 year old (amended, original protocol ≥ 55 years), have hypertension (systolic blood pressure ≥ 140 and/or diastolic blood pressure ≥ 90 mmHg), impaired fasting glucose (IFG) defined as glucose level 110–125 mg/dl in venous plasma or 100–109 mg/dl in capillary whole blood, and a glycosylated haemoglobin A1c (HbA1c) of 6–6.5% determined within the last six months.

#### Exclusion criteria

Patients were excluded if they received any antidiabetic drug treatment, had overt type 2 diabetes, fasting blood glucose level ≥ 126 mg/dl in venous plasma or ≥ 110 mg/dl in capillary blood, or casual plasma glucose concentrations ≥ 200 mg/dl, congestive heart failure, chronic renal insufficiency, history of myocardial infarction, stroke, drug or alcohol abuse, or contraindications against one of the drugs applied.

### Definitions

For definitions of normal glucose tolerance (NGT), impaired fasting glucose (IFG), impaired glucose tolerance (IGT) and overt diabetes (DM) see Table 1. *PreDiSc *Score: The score indicates the presence of pre-diabetes with a diagnostic sensivity of 78% and a specificity of 37% using the following parameters: blood pressure (BP) ≥ 140/90 mmHg, capillary fasting blood glucose ≥ 100 mg/dl (STIX) and age ≥ 55 years. Sensivity can be increased to 79% and specificity to 74% by additional determination of the HbA1c value (≥ 6%) [22].

**Table 1:** Values of plasma glucose (venous blood) for the diagnosis of diabetes mellitus* and other categories of hyperglycemia according to DDG criteria [30]

		**mg/dl**			**mmol/l**		
		**Fasting**		**2 h OGTT**	**Fasting**		**2 h OGTT**

**NGT**	**Normal glucose tolerance**	< 100		< 140	< 5.6		< 7.8
**IFG**	**Impaired fasting glucose**	100–125		-	5.6–6.9		-
**IGT**	**Impaired glucose tolerance**	< 126	and	140–199	< 7.0	and	7.8–11.0
**DM**	**Diabetes mellitus**	≥ 126	and/or	≥ 200	≥ 7.0	and/or	≥ 11.1

IGT classification according to 2 h OGG is only appropriate if the NGT value is below the threshold value for diabetes mellitus.

### Antihypertensive treatment

Patients in **Group 1 **receive Ramipril either as monotherapy (Delix^®^, Sanofi-Aventis, Berlin) or in combination with Felodipin (Delmuno^®^, Sanofi-Aventis, Berlin), patients in **Group 2 **received any other diuretic-based or β-blocker-based therapy without using ACEi or ARBs. Ramipril was chosen as the antihypertensive drug in one group, as long-standing experience from several clinical studies including large endpoint studies have accumulated with this agent [23-25]. The Heart Outcomes Prevention Evaluation (HOPE) Study showed that ramipril is effective in preventing major cardiovascular events in high-risk patients without hypertension or those whose hypertension was sufficiently controlled with other treatments [26].

Generally, treatment regimens in this study can be chosen in accordance with the recommendations of the German Hypertension League and the European Society of Hypertension [27,28] For initial treatment monotherapy or a low-dose combination regimen is suggested. If the response is inadequate, possible options include increasing the dose, changing the drug or introduction of further combination drugs [29]. All drugs are administered within the approved labelling.

Advice about the investigation has been obtained by the institutional review board of the Charité Berlin, Germany – Campus Benjamin-Franklin. Written informed consent was obtained from every patient in writing. The planned follow-up period is four years.

### Endpoints

The primary evaluation criterion of this observational study is the first manifestation of type 2 diabetes according to the current guidelines of the German Diabetes Society (see table 1). [30] Further evaluation criteria are the deterioration of pre-diabetes indicated by an increase of HbA1c of at least 10% compared to baseline value within 4 years, the initiation of antidiabetic glucose lowering medication, an increase of fasting glucose levels, change of HbA1c compared to baseline, BP reduction, achievement of the target BP <130/80 mmHg after 12 months and at the 4 year follow-up, time needed to reach target BP, major cardiovascular (CV) events (first manifestation of symptomatic coronary heart disease (CHD) and/or peripheral arterial occlusive disease and/or cerebrovascular events), type and frequency of Adverse Events (AE) or Serious Adverse Events (SAE), and total mortality. In the diuretic-based therapy group, time to switch to ACE-based or ARB-based therapy will also be analysed.

### Investigational plan

Table 2 summarizes the investigational plan. Patients are seen at 7 scheduled visits. Vital signs (BP, heart rate) will be complemented by height and weight measurements (determinations of body mass index) and waist and hip circumference measurement at after 3, 6 and 12 months and thereafter at yearly intervals. Further, lab examinations of glucose, lipids, inflammatory (high sensitive C-reactive protein) and renal parameters (with cystatin C to assess renal function [30,31]) will be done in the same intervals. Ambulatory BP monitoring is facultative and will be performed in a subset of patients. AEs will be recorded and their severity, course and relation to the medication assessed by the treating physician. Prior as well as concomitant diseases and concomitant medication will also be assessed.

**Table 2:** ADaPT study plan

**Documentation/Investigation**	**Baseline**	**12 wk**	**6 mo**	**1 yr**	**2 yr**	**3 yr**	**4 yr**
Clinical examination and medical history	x						
Information on data protection	x						
Blood pressure, heart rate	x	x	x	x	x	x	x
Physical examination: height, weight, waist and hip circumference	x			x	x	x	x
Laboratory screening (central laboratory): Blood glucose, HbA1c	x	x	x	x	x	x	x
Laboratory values: hsCRP, total cholesterol, LDL-C, HDL-C, TG, cystatin C, potassium, albumin, OGTT	x			x	x	x	x
ABPM (optional)	x			x	x	x	x
AE, SAE		x	x	x	x	x	x

Wk = weeks; mo = months; yr = years; hsCRP = high sensitive C-reactive protein; ABPM = ambulatory blood pressure monitoring; AE = Adverse Events; SAE = Serious Adverse Events

### Statistical Assumptions and sample size calculation

Sample size calculation for the primary endpoint was made under the assumption – based on the results of the ALLHAT study – that during the 4-year observational period 10.0% of patients in the ramipril-based antihypertensive regimen and 14.3% in the diuretic-based regimen will develop overt diabetes mellitus [21]. The detectable risk increase is compatible with 80% power and significance of 0.05 is 49% (OR 1,489). Based on this assumption, a sample size of *n *= 2001 was required. In terms of randomisation, addressing the literature evidence in favour of ACE inhibitor treatment, an imbalanced ratio of 2:1 for the number of patients in both treatment groups was chosen, resulting in a target inclusion number of 1334 patients in group 1 and 667 patients in group 2, respectively.

### Statistical analyses

The following parameters will be analysed: patient demographics including patient history, capillary und venous fasting glucose, HbA1c, BP, BMI, waist/hip ratio, concomitant medications, percentage of patients with prediabetes according to PreDiSc parameters [22], total cholesterol, HDL cholesterol, LDL cholesterol, triglycerides, urinary albumin, and high-sensitive C-reactive protein.

Patients treated with ramipril (and ramipril-based combination therapy) and patients who received various other antihypertensive drugs (with the exception of ACEi or ARBs) will be compared. Statistical analyses of the data will be performed as exploratory analyses. Descriptive statistics for continuous target data per treatment group and per total contain the following: number of patients, means ± standard deviation, median, minimum and maximum. The absolute and relative frequency in percentages will be determined. Per treatment group 95% confidence intervals for the means of continuous target data as well as for the relative frequency of categorical target data will be calculated using appropriate methods.

For comparison of the treatment groups with respect to the incidence of specific events (e.g. patients with first manifestation of diabetes mellitus type 2 or proportion of patients with deterioration of pre-diabetes), the chi-square or the log-rank tests will be used to compare the "survival curves". An interim analysis is scheduled after the first and second year of the observation. However, the overall significance levels will not be adjusted.

#### Baseline Characteristics

Enrolment in AdaPT started in August 2004. The last patient out of a total of 2,015 patients was included in March 2006 (table 3). 1,353 patients were enrolled into the ACEi-based group and 662 patients got a diuretic-based therapy. While age was similar between both groups the ACEi-based group had more male patients (51.4 vs. 42.6%). BMI (29.9 ± 5.0 vs. 29.8 ± 4.8 kg/m^2^) and waist-to-hip-ratio (0.95 ± 0.1 vs. 0.93 ± 0.1) were almost identical in both study groups. Dyslipidemia (56.5%), hypertension (57.8%) and overweight (43.3%) and obesity (42.6%) were the frequent baseline characteristics of patients in both groups. Baseline characteristics of AdaPT are displayed in table 3 and compared to other recent studies on the incidence of diabetes being treated with RAS blocking agents vs. conventional drugs.

**Table 3:** Baseline characteristics of the enrolled patients in AdaPT-study – compared to other trials

	**VALUE **[16]		**ASCOT-BPLA **[17]		**DREAM **[6,38]		**AdaPT**	
	**Valsartan**	**Amlodipine**	**Amlodipine**	**Atenolol**	**Ramipril**	**Placebo**	**ACEi**	**Diuretic**
	**(n = 7649)**	**(n = 7596)**	**(n = 9639)**	**(n = 9618)**	**(n = 2.623)**	**(n = 2.646)**	**(n = 1.353)**	**(n = 662)**

**Age (mean, years)**	67.2 ± 8.2	67.3 ± 8.1	63.0 ± 8.5	63.0 ± 8.5	54.7 ± 10.9	54.7 ± 10.9	67.3 ± 10.4	66.5 ± 10.2
**Male gender (%)**	57.6	57.5	77	77	40.3	41.3	51.4	42.6
**BMI (mean, kg/m^2^)**	28.6 ± 5.1	28.7 ± 5.0	28.7 ± 4.6	28.7 ± 4.5	30.9 ± 5.6	30.9 ± 5.7	29.9 ± 5.0	29.8 ± 4.8
25–29.9 kg/m^2 ^(%)	n.a.	n.a.	n.a.	n.a.	n.a.	n.a.	43.7	42.6
≥30 kg/m^2 ^(%)	n.a.	n.a.	n.a.	n.a.	n.a.	n.a.	42.4	43.2
**Waist-Hip-ratio**								
Men	n.a.	n.a.	n.a.	n.a.	0.96 ± 0.07	0.96 ± 0.07	0.99 ± 0.08	0.98 ± 0.07
Women	n.a.	n.a.	n.a.	n.a.	0.86 ± 0.08	0.87 ± 0.08	0.90 ± 0.09	0.90 ± 0.08
**History of Hypertension (%)**	92.7*	92.0*	81*	81*	34.4	43.5	56.8	59.8
**Heart rate (bpm)**	n.a.	n.a.	n.a.	n.a.	n.a.	n.a.	74.0 ± 9.4	73.0 ± 9.8
**Office systolic/diastolic BP (mmHg)**	154.5 ± 19.0/87.4 ± 10.9	154.8 ± 19.0/87.6 ± 10.7	164.1 ± 18.1/94.8 ± 10.4	163.9 ± 18.0/94.5 ± 10.4	136.1 ± 18.6/83.4 ± 10.8	136.0 ± 18.1/83.4 ± 10.8	147.4 ± 15.9/87.3 ± 9.3	144.6 ± 15.3/86.5 ± 9.4
**HbA1c (mean %)**	n.a.	n.a.	n.a.	n.a.	n.a.	n.a.	5.6 ± 0.6	5.6 ± 0.7
**Risk factors**								
Smoker (%)	n.a.	n.a.	33	32	44.1	45.0	15.6	13.6
Dyslipidemia (%)	n.a.	n.a.	n.a.	n.a.	35.6	35.4	56.4	56.6
Hyperuricemia (%)	n.a.	n.a.	n.a.	n.a.	n.a.	n.a.	21.7	23.0
MAU (%)	n.a.	n.a.	n.a.	n.a.	n.a.	n.a.	6.2	5.4
CHD (%)	45.6	46.0	n.a.	n.a.	exclusion		13.9	14.2
Stroke/TIA (%)	19.8	19.8	n.a.	n.a.	exclusion		exclusion	
**Drug therapy**								
Aspirin or antiplatelet agents (%)	n.a.	n.a.	19	19	14.3	14.3	27.7	21.9
Thiazide diuretics (%)	35.9	35.1	n.a.	n.a.	9.5	10.0	0.4	0
Nonthiazide diuretics (%)			n.a.	n.a.	5.9	5.6	0	0
ACEi	41.3	41.4	n.a.	n.a.			0	0.6
ARBs (%)	10.7	10.6	n.a.	n.a.	5.6	5.3	0.9	1.2
Betablockers (%)	32.7	33.7	n.a.	n.a.	17.2	17.5	0.3	0
CCBs (%)	41.7	40.2	n.a.	n.a.	12.8	12.9	18.6	15.3
Alphablockers (%)	7.1	6.5	n.a.	n.a.	1.9	2.2	2.1	2.0
Statins (%)	n.a.	n.a.	11**	10**	12.4	13.5	19.4	17.4
Fibrates (%)	n.a.	n.a.			2.1	2.3	0.1	0

* previously treated for hypertension; n.a. = not available; BMI = body mass index; MAU = microalbuminuria; CHD = coronary heart disease; TIA = transitory ischemic attack; ACEi = ACE inhibitors; ARBs = Angiotensin receptor blockers; CCBs = calcium channel blockers

** statins and fibrates combined.

## Discussion

Although there are several trials with a RAS based pharmacotherapy that report a reduction in the development of diabetes compared to diuretics and betablockers, these analyses were mostly post-hoc and not predefined. The only trials with a pre-defined new-onset diabetes endpoint were ASCOT-BPLA [17,18], VALUE [16] and DREAM [6] (new-onset diabetes as part of the primary endpoint). While there was a significant reduction of new-onset diabetes in ASCOT-BLPA (HR 0.70 [95%CI 0.63–0.78]) and VALUE (HR 0.77 [95%CI 0.69–0.86]) there was none in DREAM (HR 0.91 [95%CI 0.80–1.03]). Study duration was 5.5 (median), 4.2 (mean) and 3.0 (median) years. Within this setting the trial with the longest study duration had the most pronounced effect on diabetes development (see table 4).

**Table 4:** Design characteristics of AdaPT in comparison to other trials

	**VALUE **[16]		**ASCOT-BPLA **[17]		**DREAM **[6,38]		**AdaPT**	
	**Valsartan**	**Amlodipine**	**Amlodipine**	**Atenolol**	**Ramipril**	**Placebo**	**ACEi**	**Diuretic**
	**(n = 7649)**	**(n = 7596)**	**(n = 9639)**	**(n = 9618)**	**(n = 2.623)**	**(n = 2.646)**	**(n = 1.353)**	**(n = 662)**

**Study design**	RCT		RCT		RCT		Observational study	
**Endpoint**	New onset diabetes (secondary objective)		New onset diabetes (tertiary objective)		New onset diabetes or death (primary endpoint)		New onset diabetes (primary evaluation criterion)	
**Inclusion criteria**								
Age (years)	≥ 50		40–79		≥ 30		≥ 45	
Hypertension	160–210/<115 mmHg		≥ 160/100 mmHg		n.a.		≥ 140/90 mmHg	
Fasting plasma glucose	normal		Normal		110–125 mg/dl [6.1–7.0 mmol/l]		110–125 mg/dl [6.1–7.0 mmol/l]	
Impaired glucose tolerance	normal		Normal		140–199 mg/dl [7.8–11.0 mmol/l]		no inclusion criterion	
**Exclusion criteria**								
Diabetes	exclusion (for diabetes endpoint)		exclusion (for diabetes endpoint)		exclusion		exclusion	
Cardiovascular disease	possible		Possible		exclusion		possible	
**Follow-up (years)**	4.2 (mean)		5.5 (median)		3.0 (median)		4 (planned)	
**HR new-onset diabetes**	0.77 [95%CI 0.69–0.86]		0.70 [95%CI 0.63–0.78]		0.91 [95%CI 0.80–1.03]		n.a.	

n.a. = not available

Inclusion and exclusion criteria were also substantially different between ASCOT-BPLA, VALUE and DREAM. ASCOT-BPLA and VALUE included patients with treated or newly diagnosed hypertension being at least 40 years (ASCOT-BLPA) or 50 years old (VALUE). Neither impaired fasting plasma glucose nor glucose tolerance was an inclusion criterion. DREAM on the other hand included patients 30 years and above with either impaired fasting plasma glucose or glucose tolerance. Diagnosis of hypertension was not required.

### What is the additional value of AdaPT ?

The primary goal of AdaPT is to compare the effects of two antihypertensive combination therapies, an ACE inhibitor based treatment with a diuretic- (or betablocker)-based treatment on the incidence of new-onset of type 2 diabetes. To provide adequate power to discriminate a potential differential effect of these therapies, the trial is being conducted in pre-diabetic patients with hypertension and metabolic disorders in which there is a high probability for the development of diabetes.

AdaPT is conducted as an observational study in daily practice in Germany allowing for the widest possible applicability of the results obtained. This is important because there is considerable heterogeneity in patient management in daily practice and patients with pre-diabetes in clinical trials usually differ substantially from those in clinical trials. Taking the *PreDiSc *Score as a screening algorithm, the study allows physicians to effectively screen for a high risk for the development of diabetes. This would, in case of positive results, serve as an easy screening tool for high risk patients in the future.

Compared to the previously mentioned trials AdaPT included patients at a higher risk for the development of diabetes based on the selection criteria age, presence of hypertension, an impaired fasting glucose and the missing exclusion of prior cardiovascular disease. As opposed to DREAM (Placebo control) AdaPT includes patients treated with betablockers or diuretics as a control group. This further enhances the likelihood of a differential effect based on the previously mentioned data by Abuissa [32], Elliott [15] and Lam [14] (see also Figure 1). Together with the 4 year follow-up it appears likely that AdaPT may document a reduced incidence of diabetes.

### The PreDiSc Score

The fact that a score (PreDiSc) is applied in the ADaPT investigation to prospectively identify eligible patients represents a novel approach, since it shifts the conventional focus from individual risk factors (hypertension, dyslipidaemia) to a more comprehensive view that considers absolute patient risk [3,33]. To our knowledge, such an approach has only been pursued in retrospective post-hoc investigations, for example in a current analysis of the LIFE study [34].

The oral glucose tolerance test (OGTT) is the standard screening test in high risk populations (identified by medical history), but the fasting plasma glucose test is more convenient under daily practice conditions. [35] Determining fasting plasma glucose lacks sensitivity however and may miss a number of patients with diabetes. Another possible variable to determine glycaemic control is HbA1c but is less suitable for a general screening. [36,37]

Against this background, the Pre-Diabetes Score (PreDiSc) Study established a set of easy-to-determine clinical and/or laboratory parameters with close correlation to the outcomes of an OGTT [22]. Indeed, using the HbA1c alone yielded low sensitivity (58%) as did fasting glucose alone (62%). However, a combination of age ≥ 55 years, systolic BP of ≥ 140 mmHg, fasting glucose ≥ 110 – 126 mg/dl, elevated HbA1c ≥ 6% and abdominal obesity (waist circumference > 88 cm in women and 102 cm in men) was associated with high sensitivity of IGT (i.e. identification of individuals with pre-diabetes: 79%) as well as high specificity (i.e. exclusion of individuals without pre-diabetes: 74%) [22]. The low acceptance of the OGTT and the non satisfying sensitivity and/or specificity of the sole HbA1c determination speak against these parameters as inclusion criteria for studies in daily practice. In contrast, the satisfactory predictive value of the clinically easy to determine dataset evaluated in PreDiSc was the rationale to use this score as screening procedure in a prospective study. Hypertensive patients fulfilling the PreDiSc criteria have a very high likelihood to progress to overt diabetes. Compared to the original PreDiSc score, on the basis of practical experiences in the initiation phase of the study two amendments to the ADaPT protocol became necessary: first, HbA_1c _was to be measured in a central laboratory instead of the originally foreseen local laboratories (due to wide variation in locally determined values). Second, the age criterion was reduced to 45 years or older in order to accelerate the inclusion rate.

### Strength and limitations

Certain limitations of the investigation have to be taken into account: first, it is controlled, but not randomised. This means that the comparability of both cohorts can be assessed retrospectively in terms of known factors that may influence the outcomes (e.g. age, gender, comorbidity), but not in terms of unknown bias. Second, the majority of patients will require combination therapy of two, three or even more antihypertensive drugs of various classes. At clinical practice conditions, a substantial proportion of patients may receive "unallowed" medications in the course of the investigation (i.e., from the regimen of the other arm). On the other hand, the real practice conditions in this investigation convey substantial benefits that will extend the knowledge from randomised studies. Patients will be less selected than typical study patients. The dosing regimens and the combinations will reflect current use (e.g. lower doses as compared to the US for some drugs, inclusion of ARBs, etc.) and thus the results can be easily extrapolated to day-to-day clinical use.

## Conclusion

Although there are some published RCTs on the development of diabetes in patients on antihypertensive drugs, the results are inconclusive and require further investigation. The ADaPT study will provide important clinical data in a group of patients being at high risk to develop diabetes for which clear guideline recommendations regarding choice of antihypertensives are still missing.

## Conflict of interest

Dr. Paar declares to be an employee of Sanofi-Aventis Germany. All other authors have attended advisory boards and have held lectures for a number of pharmaceutical companies including Sanofi-Aventis.

## Authors' contributions

All authors have made substantial contributions to conception and design, or acquisition of data, or analysis and interpretation of data. PB has drafted the manuscript. The other authors revised the manuscript for important intellectual content. All authors have given final approval of the version to be published.
